# Exercise as an antidepressant: exploring its therapeutic potential

**DOI:** 10.3389/fpsyt.2023.1259711

**Published:** 2023-09-12

**Authors:** Dong-Joo Hwang, Jung-Hoon Koo, Tae-Kyung Kim, Yong-Chul Jang, Ah-Hyun Hyun, Jang-Soo Yook, Chang-Sun Yoon, Joon-Yong Cho

**Affiliations:** ^1^Exercise Biochemistry Laboratory, Korea National Sport University, Seoul, Republic of Korea; ^2^Sport Science Institute, Korea National Sport University, Seoul, Republic of Korea; ^3^Brain Science Institute, Korea Institute of Science and Technology (KIST), Seoul, Republic of Korea; ^4^Department of Physical Education, Korea National Sport University, Seoul, Republic of Korea

**Keywords:** depression, antidepressant, exercise, therapeutic, stress, anxiety

## Abstract

The COVID-19 pandemic has increased the prevalence of depressive disorders worldwide, requiring alternative treatments beyond medication and psychotherapy. Exercise has positive effects on the brain; therefore, it has emerged as a promising therapeutic option for individuals with depression. Considerable research involving humans and animals offers compelling evidence to support the mental health benefits of physical activity or exercise mediated by the regulation of complex theoretical paradigms. However, challenges such as conducting long-term follow-up assessments and considering individual characteristics remain in human studies despite extensive efforts. While animal studies provide valuable insights into the potential benefits of exercise and its impact on outcomes related to depression and anxiety in rodents exposed to different stress paradigms, translating the findings to humans requires careful evaluation. More research is needed to establish precise exercise prescription guidelines and to better understand the complex relationship between exercise and depressive disorders. Therefore, this concise review explores the evidence supporting exercise intervention as an antidepressant treatment and its underlying mechanisms.

## 1. Introduction

The coronavirus (COVID-19) pandemic has tremendously impacted the mental health of individuals of all ages, genders, and ethnicities. The pandemic has caused severe disruptions in everyday life, including reduced social interactions, economic strain, and increased exposure to negative information and emotions. Consequently, there has been a significant increase in the prevalence of anxiety, stress, and depression in the general population. Restrictions, such as social distancing, self-quarantine, and isolation, implemented to mitigate the spread of the disease have contributed to an increase in feelings of loneliness, despair, and isolation in people, exacerbating their depressive symptoms. Furthermore, the unpredictable nature of the pandemic, unprecedented uncertainty, and stress surrounding almost all aspects of daily life led to increased anxiety and despair (1, 2).

Depression (major depressive disorder, MDD) is characterized by persistent feelings of sadness, hopelessness, and a lack of interest. It is a serious mental illness that affects millions of people worldwide. MDD is significantly associated with various genetic, environmental, and psychological risk factors (3). Importantly, the association between stress and MDD has been of great interest to researchers, as severely stressful life events are arguably the most important risk factors for the development of depressive symptoms (4).

Several psychological and pharmacological treatments are available for MDD (4). However, many individuals with depressive symptoms do not respond to the aforementioned treatments, and some may experience unexpected side effects (5). Considering this, experts have been exploring alternative treatments for MDD, and identified physical exercise as a possible therapeutic intervention that activates multiple biological systems, such as the cardiovascular, musculoskeletal, and nervous systems. Numerous studies have investigated the effectiveness of exercise on depressive symptoms and reported that exercise can be effective in boosting mood and reducing anxiety and other mental health problems in patients with MDD (6–8).

**Figure 1:**
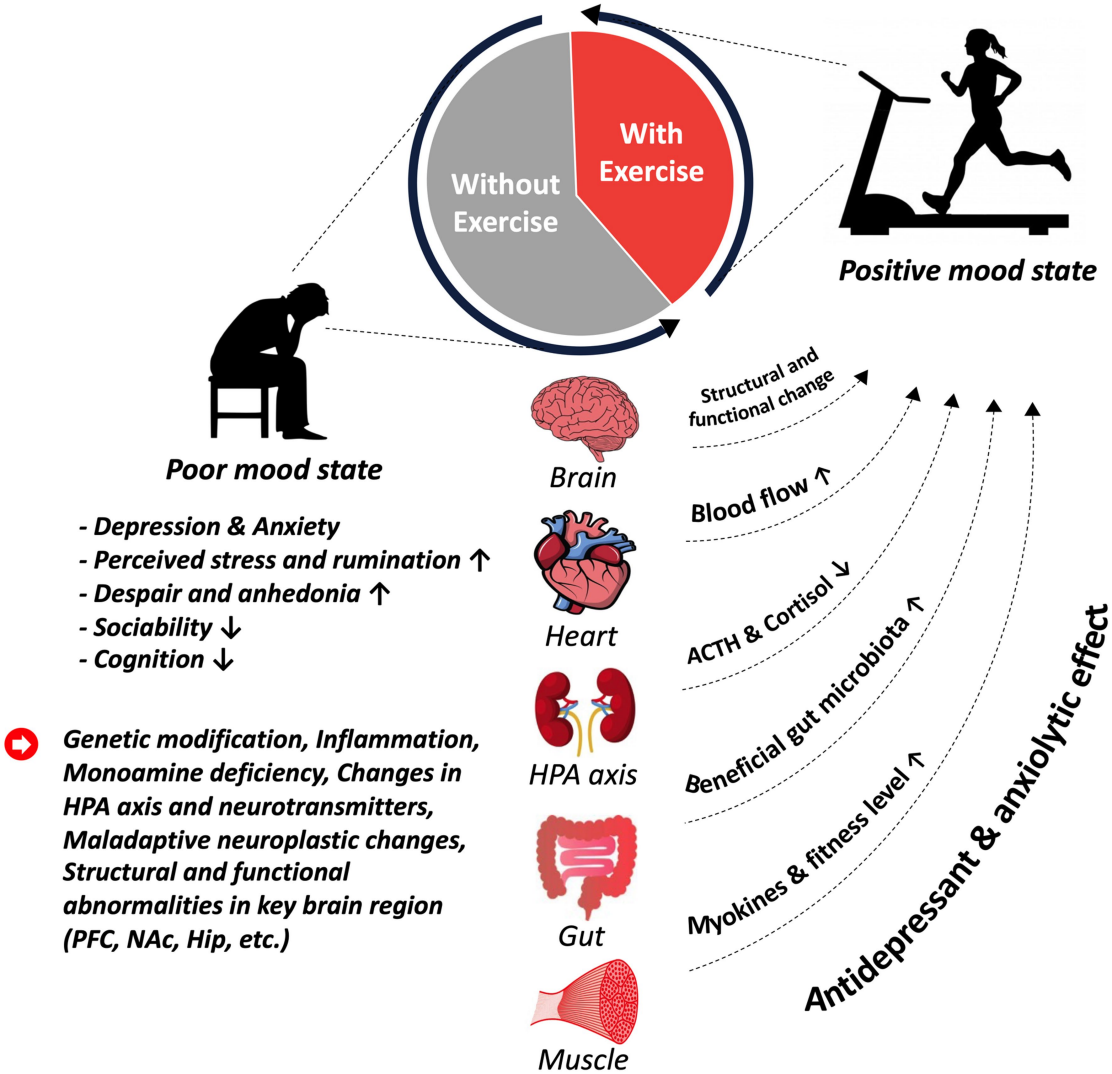
An overview of depressive disorder and the therapeutic potentials of exercise.

In previous studies, the benefits of exercise on MDD have been attributed to multiple mechanisms, including modulation (e.g., the production and release) of primary neurotransmitters, such as serotonin (5-HT), dopamine (DA), and norepinephrine (NE), which play a critical role in regulating mood (9, 10). Moreover, related studies in the field of sports science have reported a diverse range of biological mechanisms, including promoting neuroplasticity and reducing stress and inflammation, through which exercise may help alleviate depressive symptoms (10). Nevertheless, considering the nature of MDD and its heterogeneous symptoms, more research is needed to better understand the relationship between exercise and MDD and to establish the best prescription considering the optimal type, intensity, and duration of exercise for individuals with MDD. In this review, we provided an overview of the emerging scientific evidence on the effectiveness of exercise as an antidepressant treatment for MDD and discussed the underlying mechanisms by which exercise can prevent or mitigate depressive symptoms (see Figure 1).

## 2. An integrated overview of depressive disorder

Due to the etiological complexity and heterogeneity of depressive disorders, their pathophysiology remains elusive. Nonetheless, extensive studies have suggested various hypotheses underlying the pathophysiology of MDD. The most relevant neurobiological theory of MDD is the monoamine deficiency hypothesis, which suggests that depressive symptoms may be caused by a deficiency and/or imbalance in monoamine neurotransmitters such as serotonin, dopamine, and norepinephrine in the brain. From a clinical perspective, monoaminergic systems are involved in the regulation of diverse brain functions including mood, emotions, and motivation. According to related studies, low monoamine concentrations in the synaptic cleft have been observed in patients with MDD (3). Many antidepressant medications target the monoamine system to increase the availability of monoamine neurotransmitters in the nervous system. In addition, the recent development of selective serotonin reuptake inhibitors (SSRIs), serotonin-norepinephrine reuptake inhibitors (SNRIs), and tricyclic antidepressants (TCAs), as effective treatments further support the notion of monoamine deficits in MDD (11). However, the monoamine deficiency hypothesis has been challenged in recent years for oversimplification. Studies have suggested that it is unlikely that a deficient monoamine system is sufficient to manifest clinical depressive symptoms, as not every patient responds to medications, and some may experience partial or no relief in their symptoms (3, 12, 13). Accumulating evidence shows that multiple other neurotransmitter systems, including the gamma-aminobutyric acid (GABA) and glutamate systems, are associated with the pathophysiology of depressive symptoms (13). Overall, these findings suggest that the classical monoamine deficiency paradigm, which posits a straightforward correlation between neurotransmitters and depressive symptoms, may not accurately reflect the nature of MDD.

Additionally, MDD is associated with structural and functional modifications in brain circuits involved in the regulation of emotional and motivational behaviors. The brain constantly changes and adapts to everyday experiences and the surrounding environment; this is called neural plasticity. Maladaptive neuroplastic changes in patients with MDD may be caused by chronic stress responses or other negative stimuli. A recent meta-analytic study exploring the structural and functional abnormalities in MDD demonstrated a large reduction in volume and loss of dendritic spines essential for neural connections in key brain regions, such as the prefrontal cortex (PFC), nucleus accumbens (NAc), and hippocampus (HIP), which may be due to the disruption of the brain’s pro-adaptation to stress through growth and reorganization in neural networks (14, 15). Advances in neuroscience have provided further insights into the dysregulation of neurotrophic factors in the pathogenesis of MDD and anxiety. Numerous studies have revealed decreased levels of neurotrophic factors, such as brain-derived neurotrophic factor (BDNF) and its receptor tropomyosin-related kinase receptor B (TrkB), in certain brain regions, diminishing the growth of new neurons, synapses, and dendrites for development and recovery from brain damage (15–17). Thus, it is reasonable to postulate that negative alteration of neuroplasticity is a possible causative mechanism of MDD.

Moreover, scientists believe that these are not the only pathologic features that determine the possibility of MDD and other mood disorders, but there may also be a multitude of interrelated pathological mechanisms, including genetic [heritability, polygenic risk, and polymorphism (e.g., in specific factors such as the BDNF)] and environmental factors, gut microbiota, the hypothalamus-pituitary–adrenal (HPA) axis-based stress response circuit, inflammation, and other stressful life experiences; however, multiple other hypotheses are currently being explored by medical professionals and researchers (3, 18, 19). Therefore, a complete understanding of the underlying pathophysiology of depressive disorders may not be feasible. An effective control of depressive symptoms with therapeutic approaches targeting non-comorbid conditions is challenging in addition to the side effects associated with antidepressants, such as sexual dysfunction, dizziness, headaches, insomnia, and feelings of agitation or anxiety.

## 3. Exercise as a disease-modifying treatment for depressive disorder

Depressive disorders are significant public health issues affecting millions of people worldwide. Experts have suggested that advanced medical and non-medical modalities as coping strategies for MDD can help reduce the risk of developing MDD and effectively manage and treat depressive symptoms. In light of this, many human and animal studies offer compelling evidence supporting the mental health benefits of physical activity and/or exercise mediated by the regulation of complex theoretical paradigms.

Studies have consistently shown that physical exercise, defined as planned, structured, and repetitive movements to improve one or more aspects of physical fitness, is an effective treatment for depressive disorders. In the context of depressive disorder, the clinical implications of exercise interventions offer a promising avenue. The transition from preclinical findings to clinical settings, however, introduces a spectrum of considerations that require comprehensive exploration. While preclinical studies lay the foundation of treatments by elucidating their efficacy and underlying mechanisms, clinical settings introduce complexities such as diverse demographics, comorbidities, and individual variance. Thus, a detailed explanation and discussion of these complexities are needed to facilitate an effective translation by bridging the gap between controlled experiments and practical implementation (20, 21). Furthermore, it is essential to consider the potential adverse effects and dropout rates associated with exercise interventions, which might exacerbate the pre-existing conditions. Notwithstanding the foregoing, exercise interventions reportedly have almost no side effects, except in the cases where individuals face challenges related to disability and pathologic conditions upon engaging in exercise; it has also been reported by Stubbs et al. (22) and Vancampfort et al. (23) that the dropout rate for exercise intervention as a feasible evidence-based treatment in stress-related disorders, is comparable to that for control conditions (22–25).

Through an in-depth discussion of outcomes derived from rigorously conducted clinical trials, an insight into the practical implications of exercise can be attained. This, in turn, facilitates the achievement of a comprehensive and balanced perspective on the management of depressive disorder. A meta-analysis conducted by Schuch et al. (26) revealed that as a therapeutic option, exercise intervention was associated with a significant reduction in depressive symptoms and improvements in quality of life (QoL) (26). This was not simply due to a placebo effect as shown by the results in patients with MDD in the control condition. In another randomized controlled trial (RCT)-based meta-analysis, the authors found that both aerobic and resistance exercises led to clinically significant reductions in depressive symptoms in adults with MDD. These findings suggest that individuals with MDD or anxiety may benefit from choosing exercise modalities that align with their personal preferences as different types of exercise demonstrate efficacy in ameliorating depressive symptoms (27). The mechanisms underlying the benefits of physical exercise in patients with MDD are not fully understood. However, several possible conjectures may explain why exercise positively impacts mood. It is conceivable that after exercise, the brain secretes endogenous chemicals (hormones) such as β-endorphin, a natural cannabis-like chemical that helps relieve pain and boost mood, and serotonin that fosters feelings of happiness and well-being to help ease MDD and anxiety (28). Physical exercise may efficiently regulate the function of the HPA axis, which plays a critical role in the body’s stress response by regulating various physiological responses, including blood pressure, heart rate, and immune responses (9, 29, 30). Notably, the overall biological mechanisms underlying the previously reported exercise benefits are complex and remain to be elucidated.

Despite growing evidence supporting the application of physical exercise as an antidepressant for depressive disorders, it can be challenging for individuals with MDD to engage in regular exercise. Patients with MDD or anxiety may find it difficult to motivate themselves to exercise and struggle with feelings of self-doubt or low self-esteem. Therefore, it is important for healthcare providers and mental health professionals to provide support and guide individuals with depressive disorders to help them stay motivated and maintain their exercise routines.

## 4. Recent evidence on exercise as an antidepressant treatment for humans

An emerging body of evidence suggests that physical exercise leads to both structural and functional changes in the depressed brain. The impact of exercise interventions on depressive symptoms has been extensively studied as a potential treatment modality, with RCTs supporting its efficacy. The recent findings shown in Table 1 highlight the potential benefits of exercise for MDD across populations, contributing to a comprehensive body of evidence supporting exercise as an adjunctive therapy. A consistent result in scientific investigations is a significant reduction in depressive symptoms following an exercise intervention. Generally, established diagnostic criteria for severe MDD and the improvements induced by exercise treatment are verified by survey-based assessments of measures, namely the Diagnostic and Statistical Manual of Mental Disorders, Fifth Edition (DSM-5), Beck Depression Inventory (BDI), Hamilton Depression Rating Scale (HAM-D), Montgomery-Asberg Depression Rating Scale (MADRS), and Edinburgh Postnatal Depression Scale (EPSD) (41, 42). These are widely accepted classifications used by mental health professionals. Particularly, to be diagnosed with severe MDD, the individuals must meet the criteria for a major depressive episode and also exhibit specific core symptoms that indicate the severity of the disorder, a persistently depressed mood nearly every day, and a marked loss of interest and pleasure in activities. In addition to these, at least 5 or more out of the listed symptoms during a continuous period of time must be presented, which have to impact the individual’s ability to function in daily life. The diagnostic criteria are as follows (42):

- Depressed mood most of the day, nearly every day.
- Markedly diminished interest or pleasure in almost all activities (anhedonia).
- Significant weight loss or weight gain, or change in appetite.
- Insomnia or hypersomnia, nearly every day.
- Psychomotor agitation or retardation, nearly every day.
- Fatigue or loss of energy nearly every day
- Feelings of worthlessness or excessive guilt.
- Diminished ability to think or concentrate and indecisiveness, nearly every day.
- Recurrent thoughts of death, suicidal ideation, or a suicide attempt.

**Table 1:** Recent findings on the impact of exercise in individuals diagnosed with depressive disorder.

References	RD	Subject	Type (Intensity)	Duration	Primary outcomes
Philippot et al. (8)	RCTs	Adolescent inpatients with depression	Cardiovascular and strength training (45 to 59% of HRR)	6 weeks	- Depressive symptoms ↓ (HADA-D) - Anxiety → (HADA-A) - Cardiovascular capacity ↑ (VO_2_max)
La Rocque et al. (31)	RCTs	Women with a unipolar depressive disorder	Bikram yoga and aerobic exercise	8 weeks	- Depressive symptom ↓ (HAM-D) - Perceived stress and rumination ↓ (HUS and RRS)
Hidalgo et al. (32)	RCTs	Patients aged ≥65 years with a clinically significant depressive episode (MADRS)	Moderate exercise combined with aerobic and resistance training	6 months	- Depressive symptom ↓ (MADRS)
Gordon et al. (33)	RCTs	Young adults (AGAD)	Resistance exercise	8 weeks	- Anxiety symptom ↓. (STAI-Y2)
Szuhany et al. (34)	RCTs	Patient with MDD or PDD (DSM-5 criteria)	Behavior activation (BA) treatment with an adjunctive exercise	12 weeks	- Depressive symptom ↓ (MADRS and BDI-II) - BDNF level across a single exercise session ↑ - Resting BDNF level across an exercise program →
Moraes et al. (35)	RCTs	Older adults with MDD (DSM-IV criteria)	Aerobic and strength training (moderate intensity)	12 weeks	- Depressive symptom ↓ (HAM-D and BDI)
Özkan et al. (36)	RCTs	Postpartum women with depressive symptom (EPDS score)	Exercise intervention (mild to severe intensity)	4 weeks	- Postpartum depression ↓ (EPDS)
Imboden et al. (37)	RCTs	Patients with major depression (>16 of HDRS-17 score)	Aerobic exercise (indoor bicycles)	6 weeks	- Depressive symptom ↓ (HDRS and BDI) - Working memory ↑
Paolucci et al. (38)	RCTs	Normal young adults	Moderate continuous training (MCT)	6 weeks	- Depression and anxiety ↓ (BDI-II and BAI) - Proinflammatory cytokine ↓
Williams et al. (39)	RCTs	Overweight children	Vigorous aerobic exercise (activity)	8 months	- Depressive symptom → (CDI) - Fitness level ↑
Nasstasia et al. (40)	RCTs	Youth with major depression (DSM-IV criteria)	Multi-modal exercise intervention (consisting of resistance and aerobic exercise)	12 weeks	- Depressive symptom ↓ (BDI-II) - Physical (somatic) health improvements ↑ - Cognitive symptoms ↑ (BDI-II cognitive)

RD, research design; RCT, randomized controlled trial; HRR, heart rate reserve; HADS-D, The Hospital Anxiety and Depression Scale related to depression (subscale D); HADS-A, Hospital Anxiety and Depression Scale related to anxiety (subscale A); HAM-D, Hamilton rating scale for depression; HUS, Hassles and Uplifts Scale; RRS, Ruminative Responses Scale; MADRS, Montgomery-Asberg Depression Rating Scale; AGAD, analogue-Generalized Anxiety Disorder; STAI-Y2, State–Trait Anxiety Inventory for Adults (STAI) Form Y-2; DSM, Diagnostic and Statistical Manual of Mental Illnesses; BDI, Beck Depression Inventory; EPDS, Edinburgh Postnatal Depression Scale; HDRS, Hamilton Depression Rating Scale; BAI, Beck Anxiety Inventory; CDI, Children’s depression inventory.

Meanwhile, past studies have demonstrated that exercise intervention—whether moderate-to-severe aerobic and cardiovascular training, strengthening or resistance exercise, a combination of different types, or adjunctive forms of exercise—is associated with an alleviation of depressive symptoms (8, 31–37, 39, 40). Furthermore, these studies have highlighted additional positive outcomes related to MDD. Reductions in anxiety and cognitive symptoms (negative thinking and rumination), measured by anxiety rating scales such as the Beck Anxiety Inventory (BAI) and Hospital Anxiety and Depression Scale-Anxiety (HADA-A) have been observed in several studies, suggesting that exercise may have a broader positive impact on overall mood and psychological well-being (8, 33, 38). Cardiovascular benefits of exercise have also been demonstrated in several studies. Increased cardiovascular capacity, as indicated by an increase in maximal oxygen consumption (VO_2_max and VO_2_ peak), has been observed in both adolescents and young adults with MDD (8, 39). These findings are particularly noteworthy as they underscore the potential physiological benefits of exercise beyond mental health improvement. Overall, the findings discussed in these studies provide compelling evidence for the effectiveness of exercise in MDD and substantial changes in depressive symptoms, as well as in promoting physical fitness. Furthermore, the results support the inclusion of exercise intervention as an integral part of comprehensive treatment plans for MDD, alongside other evidence-based interventions such as psychotherapy and medication (see Figure 1).

Despite the valuable evidence regarding exercise for patients with depressive symptoms, it is important to acknowledge certain considerations for evaluating the effectiveness of exercise in individuals with MDD. MDD is a recurrent condition, but existing literature with different intervention periods ranging from 4 to 12 weeks lacks long-term follow-up assessment. This limits the understanding of the sustained effect of exercise intervention (40). Another important aspect to be explored in future studies is the impact of exercise on specific populations, such as older adults, children who are overweight, postpartum women, and patients with different stages of MDD (24). Although exercise has been widely accepted as an effective treatment, the demographic characteristics of the study participants, including age, sex, disease severity, presence of comorbidities, and other contextual factors, can influence its outcomes (e.g., effective response to exercise and ineffective exercise) (43). This can be accepted in the context of susceptibility, vulnerability, and resilience to stress and MDD. Therefore, the findings may not be generalizable to patients or individuals outside of the specific population.

Most studies included various types and intensities of exercise, which normally consisted of aerobic, resistance, and others. Since it can be a significant challenge for individuals with MDD to engage in and sustain an exercise regime due to low motivation, lack of energy, and reduced interest, the wide range of exercise regimes further compromises the validity and reliability of the findings and makes it challenging to draw definitive conclusions (44, 45). This inconsistency has impeded the establishment of precise exercise prescription guidelines in clinical practice. Thus, to evaluate research findings on exercise-induced antidepressant effects, mitigating the aforementioned challenges necessitates adopting standardized methodologies, conducting long-term follow-ups, and meticulously considering individual characteristics, thereby advancing our understanding of exercise as a therapeutic intervention for MDD. Considering the promising findings of exercise interventions as viable and accessible options for individuals with MDD, incorporating exercise into therapeutic plans may alleviate depressive symptoms, enhance mental well-being, and contribute to the improvement of overall health. Therefore, healthcare professionals should prioritize the integration of exercise as an adjunct element in therapeutic approaches for individuals with MDD.

## 5. Exercise-induced antidepressant effect in depression model

Animal studies offer distinct advantages in scientific research. Unlike human studies, they provide a controlled environment in which researchers can meticulously manipulate and observe specific variables of interest. Within this controlled condition, researchers are allowed to examine the effects of specific variables without interference of confounding factors (46). This precise control has provided a solid foundation for establishing the effectiveness of exercise in depressive disorders and understanding the underlying mechanisms.

Based on the latest research findings (in the last 5 years) in reputable journals (Table 2), valuable insights into the potential benefits of exercise and its impact on various outcomes related to MDD and anxiety have been demonstrated in rodents exposed to different paradigms of stress, including unpredictable stress, restraint, and social defeat stress. Antidepressant effects have also been reported in other pathological conditions accompanied by depressive and anxiety symptoms, such as diabetes, Alzheimer’s disease (AD), and Parkinson’s disease (PD) (59–62). As shown in Table 2, most studies utilized treadmill exercise (TE) and voluntary wheel running (VWR) in a research setting, which are aerobic exercises, and found that engaging in more than 2 weeks of regular exercise significantly reduced the core symptoms of depressive disorder, behavioral despair, anhedonia, and anxiety. Pagliusi et al. (54) reported a decrease in social avoidance and hyperalgesia after 32 days of VWR, suggesting that exercise not only improves mood-related behaviors, but also mitigates other social and sensory deficits associated with depressive disorders (54, 63). Both exercise modalities promote neuroplastic changes in the depressed brain. Specifically, the positive effects of TE have been attributed to increased volume and enhanced maturation of the newborn, dendritic spine density, and axonal myelination in the HIP and medial prefrontal cortex (mPFC), whereas similar effects in the HIP, basolateral amygdala (BLA), and NAc have also been demonstrated in a stress-induced depression model (49, 50, 56, 58). These structural changes in the brain subregions, which are associated with emotional processing and possibly regulating susceptibility and resilience to stress, contribute to the observed improvements in depressive-and anxiety-like behaviors (64). Furthermore, chronic neuroinflammation, a process characterized by the release of proinflammatory molecules and an immune response in the brain, has been implicated in the pathophysiology of depressive disorders, and exercise has emerged as a potential modulator. Several studies have reported a reduction in markers of microglial activation and neuroinflammation following exercise intervention, highlighting the anti-inflammatory properties of exercise as a potential antidepressant and anxiolytic mechanism (48, 52). A recent study has revealed additional avenues through which exercise interventions affect depressive disorders in rodents. Exercise-induced changes in gut microbiota composition and the gut-brain axis have been implicated as the antidepressant effects, thereby supporting the need for a comprehensive understanding of the antidepressant effects of exercise interventions (65–67).

**Table 2:** Recent findings on exercise-induced antidepressant effect in the depression model.

References	Condition of interest	Type (Intensity)	Duration	Primary outcomes
Yan et al. (47)	Anxiety (by CRS)	Treadmill exercise (not mentioned)	2 weeks (1 h daily)	- Anxiety ↓ (OFT, EPM, MBT) - Axonal myelination in mPFC ↑ - Neuronal activation ↑
Jung et al. (48)	Depression and Anxiety (by ESPS)	Resistance exercise	4 weeks (3 times per week)	- Despair and anxiety ↓ (TST, FST, EPM) - Neuroinflammation in HIP ↓
Luo et al. (49)	Depression and Anxiety (by CRS)	Treadmill exercise (at 10 m/min)	14 days	- Anxiety ↓ (OFT, EPM) - Hyperexcitation of mPFC-BLA circuit ↓
Liang et al. (50)	Depression (by CUS)	Treadmill exercise (at 5 ~ 10 m/min)	4 weeks (5 days per week)	- Behavior despair ↓ (FST, TST) - Neuroplasticity ↑ (Newborn neuron maturation and Dendritic spine in HIP)
Liu et al. (51)	Depression and Anxiety (by CUMS)	Motorized wheel running (at 5 ~ 8 m/min)	4 weeks (5 days per week)	- Anhedonia and anxiety ↓ (SPT, OFT) - Metabolomic changes in serum
Xiao et al. (52)	Depression (by CUS)	Treadmill exercise (at 10 ~ 20 m/min)	6 weeks (5 days per week)	- Anhedonia ↓ (SPT) - Microglial activation in HIP↓ - Inflammation in HIP ↓
Yoon et al. (53)	Anxiety (by CRS)	Voluntary wheel running	4 weeks	- Behavior despair and anxiety ↓ (TST, MBT, OFT) - Genetic modification
Pagliusi et al. (54)	Depression (by SDS)	Voluntary wheel running	32 days	- Social avoidance ↓ (SIT) - Hyperalgesia ↓
Wu et al. (55)	Depression (by prenatal sGC exposure)	Treadmill exercise (at 5 ~ 8 m/min)	4 weeks (5 days per week)	- Behavior despair and anhedonia ↓ (EPM, FST) - Mitochondrial dysfunction ↓
Luo et al. (56)	Depression (induced by CUS)	Treadmill exercise (at 10 ~ 20 m/min)	6 weeks (5 days per week)	- Anhedonia ↓ (SPT) - mPFC volume ↑ - Oligodendrocyte differentiation and myelination in mPFC ↑
Peng et al. (57)	Depression (by CRS)	Whole body vibration training	8 weeks (6 days per week)	- Despair, anxiety and anhedonia ↓ (FST, OFT, EPM, SPT) - Spatial learning and memory ↑ (Barnes maze) - Neuronal degeneration in HIP ↓ - Synaptic plasticity ↑ - Pathological changes in glial cells ↓
Zhuang et al. (58)	Depression (by CUMS)	Treadmill exercise (at 10 ~ 15 m/min)	3 weeks (5 days per week)	- Anhedonia and anxiety ↓ (SPT, OFT) - Changes of dendritic spine density in mPFC, BLA, HIP, and NAc

CRS, chronic restraint stress; OFT, open field test; EPM, elevated plus maze; MBT, marble burying test; mPFC, medial prefrontal cortex; ESPS, emotional single prolonged stress; TST, tail suspension test; FST, forced swim test; HIP, hippocampus; BLA, basolateral amygdala; CUS, chronic unpredictable stress; CUMS, chronic unpredictable mild stress; SPT, sucrose preference test; SDS, social defeat stress; SIT, social interaction test; sGC, synthetic glucocorticoid; NAc, nucleus accumbens.

Resistance exercise (RE), consisting of climbing a ladder for muscular and cardiovascular strengthening, and whole-body vibration training (WBV), were recently explored in depressed animals by Jung et al. (48) and Peng et al. (57), respectively; they revealed a decrease in behavioral despair and anxious behavior, along with improved neuroplasticity, neuroinflammation, and neuronal survival, supporting their use as promising therapeutic options (48, 57).

**Figure 2:**
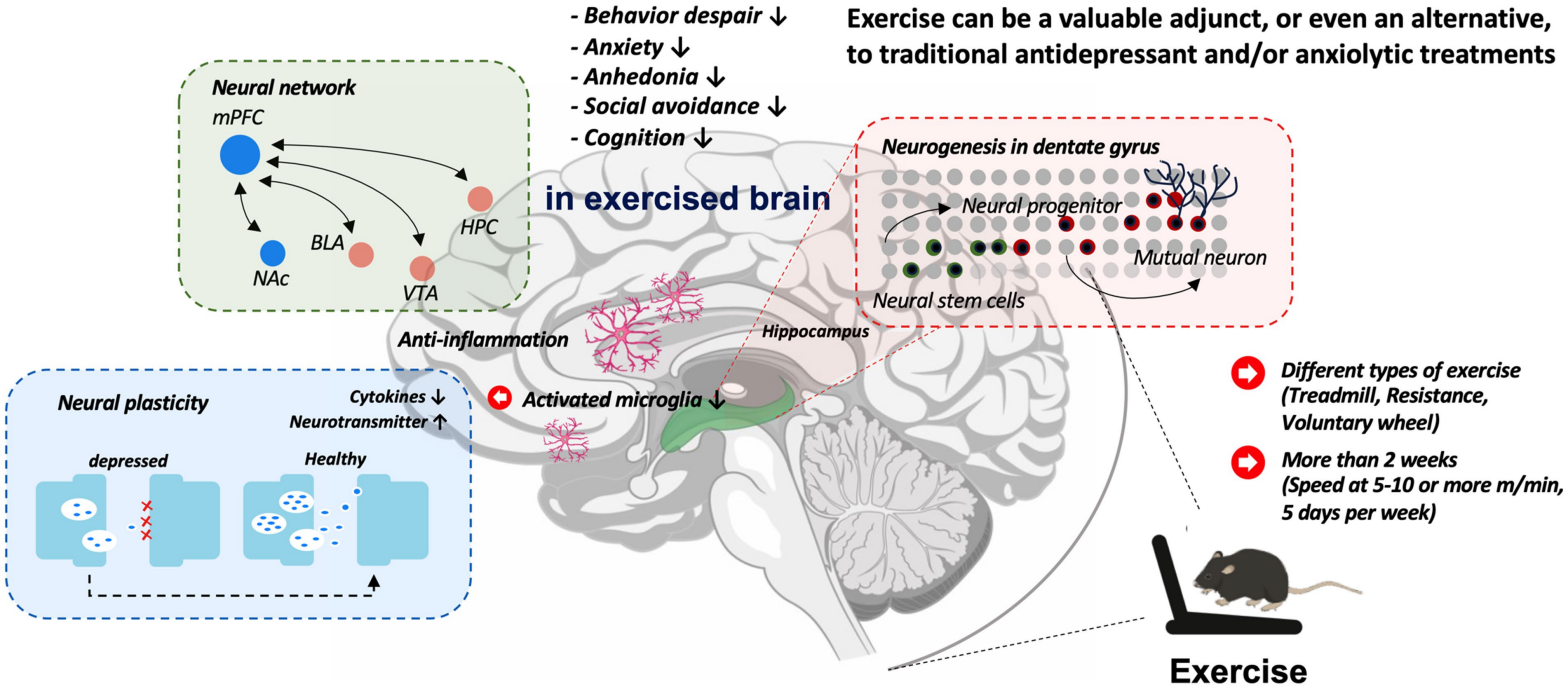
Exploring the neurobiological mechanism of exercise intervention in depression models.

The studies reviewed here collectively demonstrate the potential of different types of exercise modalities in ameliorating MDD and anxiety symptoms by counteracting the complex and multifaceted pathophysiology of depressive disorders, thereby supporting the efficacy of exercise as a non-pharmacological intervention for the management of psychiatric conditions (see Figure 2). However, it is important to recognize the limitations and complexities of translating research findings from rodents to the human population. Animal models, which are invaluable in research, have yet to fully capture the intricacies of human biology, and the underlying mechanisms in humans may differ. Nevertheless, animal studies are required to provide valuable insights that augment our understanding of the impact of exercise on MDD.

## 6. Limitations

Despite our study’s valuable insight, several limitations should be acknowledged. First, the possibility of selection bias of studies cannot be ruled out, as studies with more positive and influential outcomes are more likely to be published than those with null or negative results.

This literature/narrative review provides a general framework of summarizing and discussing the main themes, findings, and trends across various studies covering depression and exercise treatment, without necessarily conducting formal statistical analysis. This might be more interpretive and subjective on a given topic than a systemic review, which is more objective and provides a quantitative overview of previous findings. Nevertheless, the present article plays a crucial role in the academic and scientific community by helping readers understand the current state of research and potential future directions, more importantly drawing the attention of researchers working in related fields. With these limitations, it is advisable for readers to interpret this review’s findings with cautious consideration of the potential biases and variations inherent in the available studies. Lastly, given the gender difference in the incidence of depressive disorders from the gender-specific perspective, exploring the potential gender-based variations in the response to exercise intervention for depressive disorders via subsequent investigation is warranted to gain in-depth understanding.

## 7. Discussion: future perspectives and directions of exercise intervention for depression

Depressive disorders are significant public health issues. Although advanced medicine has explored coping strategies for depression, exercise has gained significant attention as a disease-modifying treatment option. Experts have suggested that exercise can be a valuable adjunct, or even an alternative, to traditional antidepressant and/or anxiolytic treatments (24, 68). Meanwhile, significant challenges of exercise treatment for individuals with MDD include difficulties in initiating and maintaining regular exercise routines due to decreased motivation, low energy level, and a lack of interest in activities. Thus, measures or treatments that enable individuals with MDD to adopt and sustain active lifestyles by themselves should be prioritized.

Despite the valuable perspectives from previous findings on exercise treatment, a lot more research is required to enhance our understanding of exercise interventions for individuals with MDD. It is necessary to conduct long-term follow-up assessments as mentioned in this review. By evaluating the sustainability of exercise benefits over an extended period, experts can determine the optimal duration and frequency of exercise interventions, leading to more effective treatment plans and maintenance strategies (40). Furthermore, to ensure consistency and comparability across exercise interventions, future research should adopt standardized methodologies. The various types and intensities of exercise used in previous studies made it difficult to expand their applicability to populations with diverse demographics. Thus, it is important to consider standardized exercise regimens to establish the validity and reliability of exercise-induced benefits, thereby enabling a more accurate exercise recommendation (24, 38).

In terms of outcomes from animal studies, there are many difficulties in translating research findings to clinical practice; nevertheless, efforts should be made to extend our understanding of the impact of exercise intervention as a therapeutic option for MDD and ultimately improve the lives of individuals with mental health problems. Conducting comparative studies involving both human and animal subjects might highlight the similarities and disparities in treatment among both populations, enabling researchers to identify the potential discrepancies in translation and gradually overcome them.

## Author contributions

D-JH: Conceptualization, Data curation, Writing-original draft, Writing - review & editing. J-HK: Investigation, Writing - review & editing. T-KK: Investigation, Writing - review & editing. Y-CJ: Investigation, Writing - review & editing. A-HH: Data curation, Investigation. J-SY: Data curation, Visualization, Writing - review & editing. C-SY: Writing - review & editing. J-YC: Supervision, Writing - review & editing.

## Funding

The author(s) declare financial support was received for the research, authorship, and/or publication of this article. This work was supported by the Ministry of Education of the Republic of Korea and the National Research Foundation of Korea (NRF-2022S1A5B5A16052533 to D-JH) and Basic Science Research Program through the National Research Foundation of Korea (NRF) funded by the Ministry of Education (NRF-2022R1I1A1A01070927 to D-JH).

## Conflict of interest

The authors declare that the research was conducted in the absence of any commercial or financial relationships that could be construed as a potential conflict of interest.

## Publisher’s note

All claims expressed in this article are solely those of the authors and do not necessarily represent those of their affiliated organizations, or those of the publisher, the editors and the reviewers. Any product that may be evaluated in this article, or claim that may be made by its manufacturer, is not guaranteed or endorsed by the publisher.

## Figures and Tables

**Table:** 

MDD	major depressive disorder
SSRIs	serotonin reuptake inhibitors
SNRIs	serotonin-norepinephrine reuptake inhibitors
TCAs	tricyclic antidepressants
GABA	gamma-aminobutyric acid
PFC	prefrontal cortex
NAc	nucleus accumbens
HIP	hippocampus
BDNF	brain-derived neurotrophic factor
TrkB	tropomyosin-related kinase receptor B
HPA	hypothalamus-pituitary–adrenal
RCT	randomized controlled trial
DSM-5	Diagnostic and Statistical Manual of Mental Disorders, Fifth Edition
BDI	Beck Depression Inventory
HAM-D	Hamilton Depression Rating Scale
MADRS	Montgomery-Asberg Depression Rating Scale
EPSD	Edinburgh Postnatal Depression Scale
BAI	Beck Anxiety Inventory
HADA-A	Hospital Anxiety and Depression Scale-Anxiety
TE	treadmill exercise
VWR	voluntary wheel running
mPFC	prefrontal cortex
BLA	basolateral amygdala
WBV	whole-body vibration training
